# Del Río Hortega’s insights into oligodendrocytes: recent advances in subtype characterization and functional roles in axonal support and disease

**DOI:** 10.3389/fnana.2025.1557214

**Published:** 2025-03-12

**Authors:** Eneritz López-Muguruza, Carla Peiró-Moreno, Fernando Pérez-Cerdá, Carlos Matute, Asier Ruiz

**Affiliations:** ^1^Department of Neurosciences, University of the Basque Country UPV/EHU, Leioa, Spain; ^2^Achucarro Basque Center for Neuroscience, Leioa, Spain; ^3^CIBERNED-Instituto de Salud Carlos III, Leioa, Spain; ^4^Department of Neurosciences, Biobizkaia, Barakaldo, Spain

**Keywords:** oligodendrocyte, myelin, axon, metabolism, disease

## Abstract

Pío Del Río Hortega (1882–1945) was a giant of modern neuroscience and perhaps the most impactful member of Cajal’s School. His contributions to clarifying the structure of the nervous system were key to understanding the brain beyond neurons. He uncovered microglia and oligodendrocytes, the latter until then named mesoglia. Most importantly, the characterization of oligodendroglia subtypes he made has stood the omics revolution that added molecular details relevant to comprehend their biological properties. Astounding as it may seem on today’s eyes, he postulated a century ago that oligodendrocytes provide trophic support to axons, an idea that is now beyond doubt and under scrutiny as dysfunction at the axon-myelin unit is key to neurodegeneration. Here, we revised recent key advancements in oligodendrocyte biology that shed light on Hortega’s ideas a century ago.

## 1 Introduction: Pío Del Río Hortega’s legacy and the metabolic roles of oligodendrocytes

Pío del Río-Hortega (1882–1945) was one of the most prominent figures in the Spanish school of neurology (Boullerne and Feinstein, 2020; De Castro, 2019; Del Río-Hortega Bereciartu, 2020; Pérez-Cerdá et al., 2015). Mentored by the exceptional neurohistopathologist Nicolás Achúcarro, he succeeded him as the head of the “Laboratory of Neuropathology,” a division of Cajal’s laboratory. There, he revolutionized the study of glial cells by discovering microglia and oligodendrocytes (OLs) through innovative modifications of silver staining techniques (Río-Hortega, 1917). For the first time, his method successfully stained distinct cytoplasmic projections in apolar cells, previously classified as the “third element.” He identified two distinct cell types and clarified their origins: microglia, the true “third element” due to its mesodermal origin, and the “interfascicular glia,” characterized by very few processes, which he grouped with astrocytes as part of the “second element” due to their shared ectodermal origin (Río-Hortega, 1920). Shortly after, he coined the term “oligodendroglia” (oligo = few; dendro = branches) for interfascicular glia, as these cells were found not only in white matter but also diffusely distributed throughout the Central Nervous System (CNS), often clustered near neurons (Río-Hortega, 1921).

His findings on microglial structure, its surveillance function, and its phagocytic capacity were relatively quickly accepted by the scientific community, although the mesodermal origin of microglia remained a subject of controversy. In contrast, his discovery of oligodendroglia faced skepticism from his contemporaries, delaying its acceptance and ultimately contributing to his dismissal from Cajal’s laboratory. Undeterred, del Río-Hortega established his own laboratory, where he mentored several distinguished scientists, including Wilder Penfield. Throughout the 1920s, he conducted extensive research on this newly identified cell type. In 1928, he published a comprehensive monograph detailing the morphology and function of oligodendroglia (Río-Hortega, 1928). By this time, he had developed a new metal impregnation protocol based on the Golgi method, known as the Golgi-Hortega technique, which provided unprecedented morphological detail. He classified OLs into three types based on their spatial relationships: interfascicular OLs (aligned in rows along axonal tracts), perineuronal OLs (closely associated with neuronal somata), and perivascular OLs (adjacent to blood vessels but lacking direct contact). Del Río-Hortega was fascinated by the intricacy, richness, and diversity of oligodendrocytic morphology. Based on variations in soma size and shape, the number and orientation of cellular processes, their distribution within the CNS, their interactions with axons, and the size of individual axons, he further categorized OLs into four subtypes, while acknowledging the absence of strict boundaries between them.

This classification was not merely descriptive; del Río-Hortega also integrated morphological and physiological insights into oligodendroglia, laying the foundation for the concept of neuroglioma (Río-Hortega, 1942). He proposed that OLs maintain a close association with neurons and hypothesized that they serve mechanical, trophic, and myelinogenic functions. Throughout his career, he gathered substantial evidence supporting the role of OLs in myelin formation, either directly or by supplying axons with essential materials. However, he remained cautious in drawing definitive conclusions—a confirmation that ultimately came with the advent of electron microscopy in the 1960s. This temporal gap, along with the limitations of oligodendroglia staining techniques prior to the introduction of immunohistochemistry, contributed to the under appreciation of his work. Additionally, many of his seminal publications were written in Spanish (Iglesias-Rozas and Garrosa, 2012), further limiting their international recognition compared to his discovery of microglia. Despite these challenges, del Río-Hortega was twice nominated for the Nobel Prize. However, he was never awarded the honor, largely due to extrinsic factors such as the hostility and envy of certain conservative Spanish academics, as well as the political climate of the time—he spent the final years of his life in exile (Del Río-Hortega Bereciartu, 2020).

The OL phenotypic diversity proposed by del Río-Hortega was initially neglected, and confirmed later with observations arising from different regions of gray and white matter, ultrastructural analysis by electron microscopy, intracellular dye injection, size and shape morphometric analysis as well as electrophysiology studies and molecular markers (Lassmann, 2012; Butt, 2013; van Bruggen et al., 2017; Edgar et al., 2021). In addition, recent evidence about axonal metabolic support provided by OLs (Asadollahi et al., 2024) relates to the concept of neurogliona suggested by Río-Hortega (1942).

## 2 Modern characterization of oligodendrocytes: a molecular perspective

The last decade has witnessed an enormous advance in the characterization of oligodendroglia. It is now well established that OL populations also vary in the way they generate myelin in terms of internodal length and sheath thickness (Bechler et al., 2015) and that OL progenitors differ in their properties during development and in the mature CNS (Crawford et al., 2016). Most notably, transcriptomics has uncovered distinct gene expression profiles and functional characteristics of these subtypes, highlighting their diverse metabolic functions and interactions with neurons. In this section, we focus on the classification arising from transcriptome analysis and specify new features recently uncovered of both perivascular and disease-associated oligodendroglia.

### 2.1 Transcriptome analysis unveiled unexpectedly diverse oligodendrocyte subtypes with distinct molecular signatures

Microarray analysis provided a deeper insight into oligodendroglia diversity. Thus, Barres and colleagues provided the first glimpse of the different and complex transcriptomic signatures of mouse OL progenitors as well as newly-formed and myelinating OL from different areas (Cahoy et al., 2008; Zhang et al., 2014). Subsequently, microarray studies clarified the expression profiles of OL progenitors in demyelination and remyelination (Moyon et al., 2015).

Although microarray and bulk RNA sequencing provided substantial information about oligodendroglia subtypes, they lacked single cell and spatial resolution. Indeed, it was with the introduction of single cell/nuclei RNA sequencing that we have drastically refined our understanding of OL subtypes and revealed their heterogeneity and new specific roles in the CNS (Marques et al., 2016; Weng et al., 2025; Zeisel et al., 2015). In this regard, the pioneering work by Castelo-Branco’s laboratory has been enlightening and highly clarifying (Castelo-Branco et al., 2024; van Bruggen et al., 2017).

**Figure 1:**
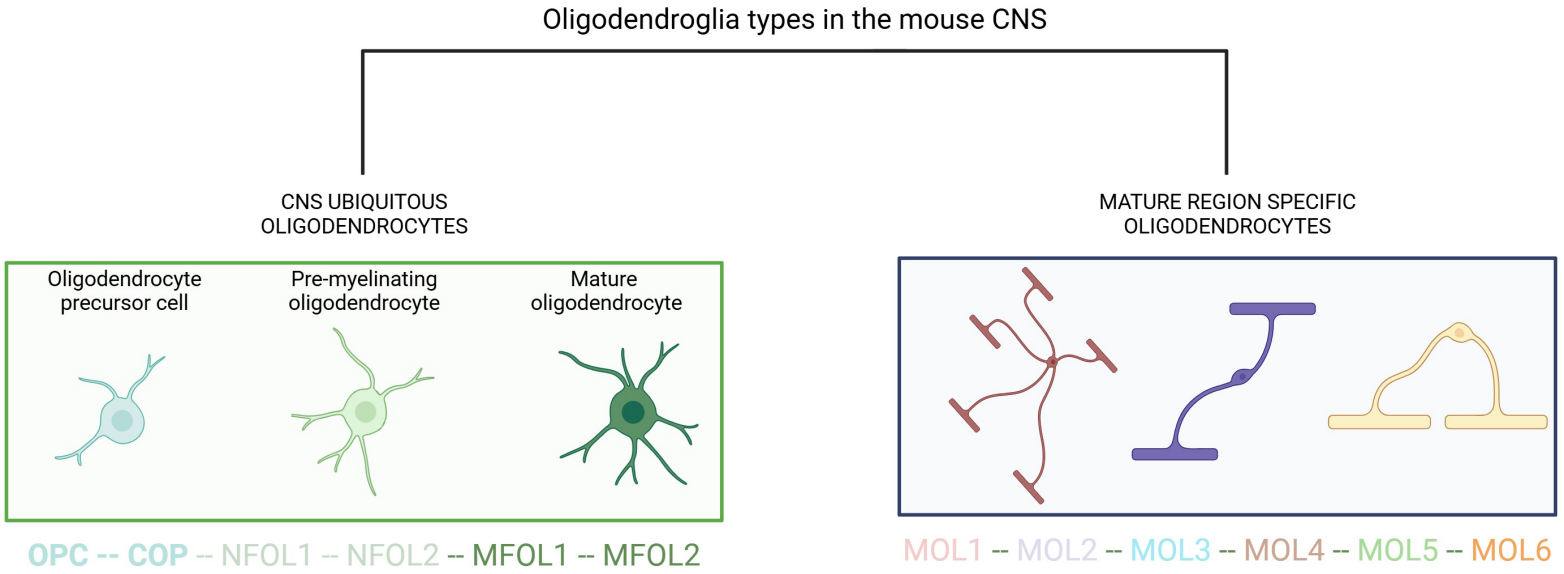
Current view of oligodendroglia heterogeneity. OPC, oligodendrocyte precursor cell; COP, committed oligodendrocyte progenitors; NFOL, newly formed oligodendrocyte; MFOL, myelin-forming oligodendrocyte; MOL, mature oligodendrocyte. Adapted from van Bruggen et al. (2017).

Single-cell RNA sequencing of the OL lineage in the mouse juvenile and adult CNS identified twelve distinct populations of OLs which represent a continuum from PDGFRα (+) OL precursor cells (OPCs) to distinct mature OLs (Marques et al., 2016) (Figure 1). This study also unveiled a second PDGFRα (+) population, distinct from OPCs, located along blood vessels. Interestingly, newly formed OLs in the adult CNS respond to complex motor learning. These rapidly myelinating OLs may also participate in remyelination in disease. Newly formed OLs are characterized by the expression of inositol 1,4,5-trisphosphate receptor type 2 (ITPR2), an endoplasmic reticulum calcium channel, revealing the key role of calcium homeostasis in oligodendroglia maturation (Zeisel et al., 2015). The main determinants of the distinct molecular signatures in each oligodendrocyte subtype and their functional correlates are described in detail in the references in this section.

### 2.2 Perivascular oligodendroglia contribute to the microvasculature environment

Del Río-Hortega’s first description of oligodendroglia postulated a possible functional connection between mature OLs and the vasculature (Río-Hortega, 1928). Indeed, OPCs migrate along blood vessels during development and repair while exchanging signals that promote differentiation and survival as well as stimulating angiogenesis (Tsai et al., 2016). At the same time, they contribute to blood–brain barrier (BBB) integrity promoting tight-junction protein expression in endothelial cells (Seo et al., 2014). Likewise, the latter favour myelin formation by mature OLs (Swire et al., 2019) that in turn release metalloproteinases contributing to vascular remodelling. These interactions appear to occur throughout the CNS as they take place in hippocampus, cerebral cortex and overall, in white and gray matter across different species. Specifically, around a fifth of mature OLs contact with capillaries, arterioles and venules in the cerebral cortex, hippocampus and cerebellar cortex (Palhol et al., 2023). This intimate interaction involves direct connection with the vascular basement membrane and possibly metabolite exchange with endothelial cells that may favour repair mechanisms during remyelination (Palhol et al., 2023). Therefore, perivascular oligodendroglia may indeed be an integral part of the vasculature microenvironment and contributes to the properties of the BBB.

### 2.3 Disease-associated oligodendroglia: bridging the gap between inflammation and neurodegeneration

Single-cell/nuclei RNA sequencing and spatial transcriptomics revealed disease-associated states of microglia and astrocytes changing the classical view of these cell types of merely two alternative states in health and disease respectively, to a more elaborated view in which both cell types undergo a gradient of transitional stages (Escartin et al., 2021; Paolicelli et al., 2022). Unexpectedly, emerging data indicates that oligodendroglia may also experience gradual changes during inflammation in multiple sclerosis (MS) (Falcão et al., 2018). Indeed, one could term those states as inflammatory OLs opening the gate to the idea that these cells may become immunocompetent under certain conditions (e.g., encephalomyelitis and upon IFN-*γ* exposure) as they may express MHC-I and –II genes and thus, contribute to neuroinflammation and neurodegeneration (Castelo-Branco et al., 2024; Kirby and Castelo-Branco, 2021).

**Figure 2:**
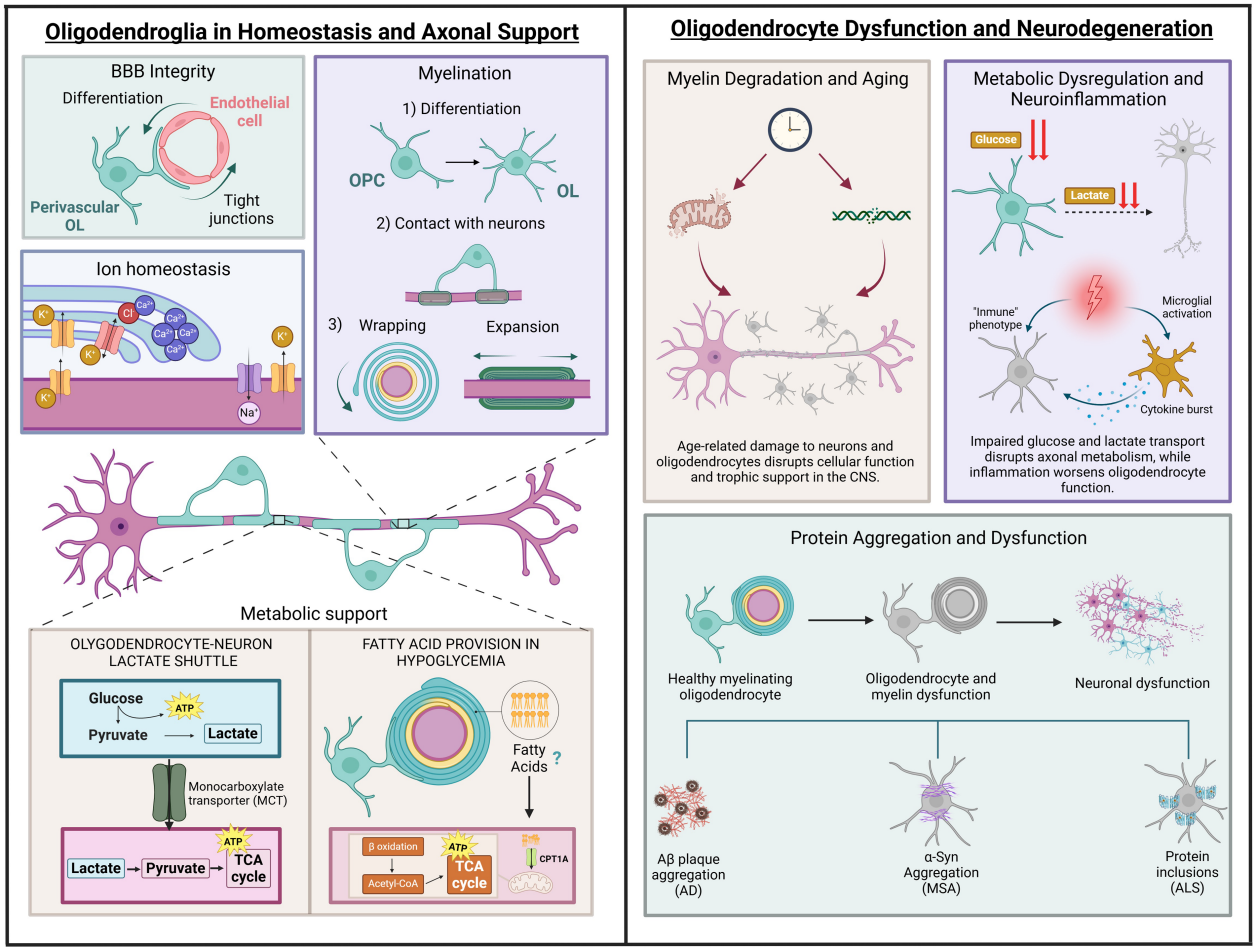
Roles of oligodendrocytes in health and disease. The left section depicts physiological functions, including blood–brain barrier support, ion homeostasis, myelination and metabolic support through glucose and lactate transport, as well as fatty acid provision during hypoglycaemia. The right section illustrates pathological processes: (1) Age-related changes, represented by thinning myelin, disrupted axon-oligodendrocyte support, and markers of DNA and mitochondrial dysfunction due to aging. (2) Metabolic breakdown, highlighting disrupted glucose and lactate transport, mitochondrial dysfunction, and neuroinflammation from activated microglia and cytokines. (3) Protein aggregation in neurodegenerative diseases, showing the progression from healthy oligodendrocytes to dysfunction and neuronal degeneration, linked to amyloid plaques (AD), α-synuclein (MSA), and protein inclusions (ALS). Illustration created with BioRender.com.

This novel idea provides solid evidence that oligodendroglia, in addition to their myelinating properties, have the potential to transition to disease-associated states, characterized by the expression of immune genes in the inflamed CNS (Castelo-Branco et al., 2024). Thus, inflammatory cytokines secreted during inflammation may drive secretion of chemokines and expression of MHC-I in OL progenitors that might propagate the immune response. The fate of activated progenitors is unclear. Evidence suggests alternative possibilities including cell death via caspase 3/7 upregulation, survival maintaining their disease-associated state, or return into homeostatic progenitors to promote remyelination. In addition, inflammation can also turn mature OLs into a disease stage along with MHC-I/II expression using similar mechanisms to those in progenitors (Figure 2).

## 3 Oligodendrocyte metabolism and axonal support

OLs, traditionally recognized as the primary architects of myelin in the CNS, are now also acknowledged for their role in sustaining axonal health through metabolic support (Baumann and Pham-Dinh, 2001). While their contribution to forming and maintaining the myelin sheath for axonal insulation has been understood since the early 1920s, supported by numerous studies and electron microscopy techniques (Bunge et al., 1962; Río-Hortega, 1928; Doyle, 1978), their functions extend beyond this structural role. In addition to facilitating saltatory conduction for efficient nerve impulse transmission, OLs are essential components of a broader glial network that provides metabolic support and regulates the neuronal environment through ion and water homeostasis (Nave, 2010; Stadelmann et al., 2019). This ion balance is regulated by channels located at the junction of the myelin sheath and the axon (Suminaite et al., 2019). As a key signalling molecule and second messenger, the regulation of intracellular calcium concentration ([Ca^2+^]_i_) in OLs, including specific regions within the myelin sheath, plays a crucial role in myelin formation and remodelling. It may also affect other functions that are yet to be fully uncovered (Paez and Lyons, 2020). Building on this understanding of OL functions is essential to examine how these cells meet their high-energy demands through specific metabolic processes.

### 3.1 Metabolic pathways in oligodendrocytes: glucose, lactate, and ketones

Glucose metabolism plays a critical role in the CNS, serving as the primary energy source for brain cells and supporting vital functions such as ATP production, neurotransmitter synthesis, and oxidative stress regulation (Mergenthaler et al., 2013). The brain generates ATP mainly through two metabolic pathways: glycolysis, which occurs in the cytosol, and oxidative phosphorylation (OXPHOS) in the mitochondria (Dienel, 2019). In comparison to glycolysis, OXPHOS produces more ATP due to the involvement of the citric acid cycle (TCA). Although the TCA cycle itself does not produce significant ATP, it provides electrons to the electron transport chain (ETC). The ETC then generates a mitochondrial membrane potential that drives ATP production through OXPHOS, with oxygen serving as the final electron acceptor (Arnold and Finley, 2023; Bonora et al., 2012).

Despite their high-energy demands, due to involvement in energy-intensive functions such as myelin maintenance, protein and lipid synthesis, and cytoskeleton remodelling, OLs exhibit a preference for glycolysis over OXPHOS to meet their ATP needs (Narine and Colognato, 2022) (Figure 2). This reliance on glycolysis not only supports their energy needs but also enables them to function efficiently in the low-oxygen environment of the white matter, where OLs are present in significant numbers. Moreover, mature OLs can metabolize glucose in the cytosol and pyruvate in mitochondria (Amaral et al., 2016). The transition to glycolysis following developmental myelination indicates that OLs adopt a strategy of decreasing reliance on mitochondrial energy metabolism to support myelin maintenance and axonal integrity (Fünfschilling et al., 2012). This shift from OXPHOS to glycolysis may help minimize the production of reactive oxygen species (ROS), which is often associated with active ATP synthesis (Rosko et al., 2019).

OLs obtain glucose from the bloodstream through the uptake of the glucose transporter GLUT1, which is expressed both in the endothelial cells of the BBB and on the outer membrane of the OLs themselves (Philips and Rothstein, 2017; Simpson et al., 2007). Once glucose enters the cell, it can be utilized in various biochemical pathways or stored as glycogen, as seen in astrocytes (Lee et al., 2021), though it is unclear whether oligodendrocytes store glycogen. The glycolytic pathway generates pyruvate as its end-product. Pyruvate can either be transported into the mitochondria and converted to acetyl-CoA for entry into the citric acid cycle or be reduced to lactate by lactate dehydrogenase (Philips and Rothstein, 2017; Zangari et al., 2020). One way in which OLs provide metabolic support to axons is by exchanging glucose metabolic derivatives through various monocarboxylate transporters (MCTs) once they have been metabolized (Nave et al., 2023).

OLs express MCT1 in their myelin, which selectively transports lactate to neurons, where it is taken up via MCT2 (Lee et al., 2012), a process referred to as the oligodendrocyte-neuron lactate shuttle (Tepavčević, 2021). Disruption of this shuttle, such as through conditional deletion of MCT1 in OLs, results in significant axonal injury and motor neuron death in animal models (Philips et al., 2021). Moreover, a deficiency in either MCT1 or MCT2 leads to axonal degeneration in brain slices. Interestingly, only the loss of MCT1 can be rescued by exogenous L-lactate, as MCT2 facilitates the direct transport of lactate into axons (Pepper et al., 2018).

Similarly, ketone bodies, which act as an alternative energy source for various cell types, are exported by endothelial cells and taken up by OLs and astrocytes via MCT1, and by neurons through MCT2, owing to their monocarboxylate structure (Jensen et al., 2020; Lee et al., 2012). Among them are acetoacetic acid and *β*-hydroxybutyrate, which are primarily produced from fatty acids in the liver, though astrocytes also contribute to their synthesis (Fernandes et al., 2025). Once inside the cell, ketone bodies can be converted into acetyl-CoA, similar to pyruvate, and enter the TCA cycle to fuel OXPHOS for high ATP yield (55,56). Alternatively, ketone bodies may be used for lipid synthesis and myelin production. Specifically, OPCs and OLs can utilize ketone bodies for myelin lipid synthesis, with a potential role in ATP generation, particularly during active developmental myelination (Koper et al., 1981; Poduslo and Miller, 1991; Tepavčević, 2021).

### 3.2 Insights into lipid metabolism

To understand further the role of lipid metabolism in OLs beyond myelin recycling, it is important to delve deeper into how fatty acid *β*-oxidation and autophagy coordinate energy management and myelin integrity. Autophagy, particularly the autophagy-lysosomal pathway, not only facilitates the recycling of lipids from myelin degradation but also helps to regulate the internal lipid stores of OLs. Due to the high lipid demand required for myelination, this step is believed to be crucial in OL function (Aber et al., 2022; Belgrad et al., 2020).

Fatty acids undergo β-oxidation, a series of enzymatic degradations in the mitochondria that produce acetyl-CoA to support OXHPOS, providing energy for metabolically demanding functions (Houten and Wanders, 2010). Emerging research indicates that fatty acids can act as an alternative energy source when glucose is scarce. Both OLs and astrocytes may utilize fatty acids to generate energy or transfer metabolites to axons, thereby supporting neuronal function (Lee et al., 2021; Mi et al., 2023; Nave et al., 2023). In Drosophila, research shows that when glycolysis is compromised, glial cells depend on mitochondrial fatty acid breakdown and ketone body production to sustain neuronal function. This study underscores that during extended periods of starvation or glucose deprivation, glial fatty acid metabolism is vital for maintaining neuronal energy and fly survival. Additionally, glial cells serve as metabolic sensors, mobilizing lipid reserves to maintain brain energy balance (McMullen et al., 2023; Schulz et al., 2015).

In an *ex vivo* study of optic nerves, OLs showed notable resilience to glucose deprivation. Indeed, during low glucose conditions, axonal ATP and action potentials depend on fatty acid β-oxidation. OLs appear to provide an energy reserve for white matter and protect axons from conduction blocks, although they do not support high frequency spiking. Disruption of GLUT1 in OLs results in myelin imbalance and gradual demyelination, suggesting that impaired myelin regulation may contribute to myelin thinning in aging and disease. These findings highlight the critical role of oligodendroglial lipid metabolism in maintaining brain function during energy shortages (Asadollahi et al., 2024).

Furthermore, peroxisomes, small organelles in eukaryotic cells involved in metabolism and detoxification of ROS, contribute to lipid metabolism by processing very long chain fatty acids, similar to mitochondria (Kassmann et al., 2007). These organelles are abundant in the CNS, particularly in glial cells, and are found in the inner regions of the myelin sheath (Kumar et al., 2024; Richert et al., 2014). Disruption of peroxisomal proteins, known as peroxins, can lead to white matter abnormalities, suggesting a key role for peroxisomes in axonal support and myelin maintenance (Kassmann, 2014).

## 4 Dysfunction at the axon-myelin unit and neurodegeneration

The extraordinary energy demands of the brain render it particularly vulnerable to bioenergetic disruptions (Figure 2). Long, myelinated axons are especially affected by defective energy metabolism due to their high energy requirements associated with transmission and other essential maintenance processes. Given that OLs play a crucial role in the trophic support of myelinated axons, bioenergetic dysfunction in these cells has become the focus of numerous recent studies on neurodegeneration. In MS, inflammation causes a selective loss of OLs that ultimately leads to demyelination, axonal damage, and neurodegeneration. Consequently, oligodendroglial dysfunction has assumed a central role in the study of this disease (Trapp and Nave, 2008). Metabolomic studies have revealed that patients with MS exhibit numerous alterations in markers related to glucose, lactate, and fatty acid metabolism in cerebrospinal fluid (López-Muguruza and Matute, 2023). Additionally, in progressive MS lesions, a state of virtual hypoxia occurs due to defects in microcirculation and the release of toxins that interfere with energy metabolism (Lassmann, 2003), which is consistent with oligodendroglial metabolic dysfunction. Under these metabolic stress conditions typical of MS lesions, studies in human OLs have shown that these predominantly glycolytic cells reduce their metabolic rate, prioritizing their own survival at the expense of trophic support to the axon (Rone et al., 2016). This phenomenon may constitute a mechanism of axonal damage preceding the retraction of myelin, a hallmark of MS (Metz et al., 2007). Furthermore, human OPCs, also predominantly glycolytic but metabolically more active, did not reduce their metabolism under metabolic stress conditions, which becomes toxic for these cells and hinders remyelination (Rone et al., 2016). On the other hand, the high energy demands for the synthesis and maintenance of myelin compel OLs to utilize mitochondrial OXPHOS for ATP production as a complement to glycolysis (Harris and Attwell, 2012). Additionally, defects in mitochondria contribute to axonal damage and neurodegeneration in MS. Some characteristic mitochondrial alterations observed in white matter lesions include defects in the machinery responsible for OXPHOS and mtDNA, leading to oxidative stress, among others (Witte et al., 2019). Furthermore, in an animal model of MS, such as experimental autoimmune encephalomyelitis (EAE), alterations such as mitochondrial depolarization and reduced expression of complex I of the respiratory chain precede demyelination and axonal degeneration (Sadeghian et al., 2016). However, evidence regarding whether mitochondrial dysfunction in MS occurs within OLs themselves or in other components of white matter, such as astrocytes and axons, remains limited. The finding that human hereditary mitochondrial diseases, such as Leber’s hereditary optic neuropathy (LHON), cause oligodendroglial cell death and demyelination (78) and increase the risk of developing MS (Vanopdenbosch et al., 2000) supports this notion. For instance, Mahad et al. (2008) identified defects in complex IV of the respiratory chain in OLs within ischemic active lesions (Mahad et al., 2008). More recently, a study conducted in animals with EAE identified oxidative stress and alterations in mitochondrial morphology in oligodendrocytes prior to the infiltration of inflammatory cells (Steudler et al., 2022). Additionally, double-strand breaks in mtDNA in mice induce mitochondrial dysfunction and apoptosis in OLs, leading to subsequent demyelination (Madsen et al., 2017).

On the other hand, neurodegenerative diseases associated with aging share a key element: the progressive loss of neurons as a central trigger of their pathogenesis. Furthermore, bioenergetic dysfunction and glucose hypometabolism are characteristics common to Alzheimer’s disease (AD), Parkinson’s disease (PD), amyotrophic lateral sclerosis (ALS), and Huntington’s disease (HD), among others (Yang et al., 2023). However, with aging, white matter is affected as much as gray matter (Gunning-Dixon et al., 2009), and dysfunction of the axon-myelin coupling has emerged as a potential neuropathological mechanism in numerous neurodegenerative diseases. In AD, the leading cause of dementia and the most prevalent neurodegenerative disorder, significant damage is observed in the white matter of postmortem brains (Gouw et al., 2008; Nasrabady et al., 2018), even in the preclinical stages of the disease (Dean et al., 2017; Hoy et al., 2017), along with numerous pathological changes in the expression of oligodendroglial genes related to myelination (Mathys et al., 2019). This and other evidence support the notion that OLs not only respond to pathology but may also contribute causally to AD (Bartzokis, 2011; Han et al., 2022). AD is characterized at the molecular level by extracellular deposits of *β*-amyloid (Aβ) and intracellular accumulation of phosphorylated tau; however, the exact role of these aggregates in the onset of the disease has yet to reach a consensus within the scientific community. One of the most studied hypotheses is the amyloid cascade hypothesis, which posits that the accumulation of soluble oligomers of Aβ triggers the disease (Selkoe, 2008). Interestingly, amyloidosis is significantly increased in animal models of familial AD that also carry oligodendrocyte-specific mutations that impair to myelin integrity (Depp et al., 2023). These findings suggest a causal relationship between oligodendroglial dysfunction and Aβ deposits (Nave et al., 2023) and that disruption of oligodendrocyte-axon coupling severely compromises trophic support to axons in AD, promoting Aβ accumulation due to defects in axonal transport and lysosomal degradation. In turn, the accumulation of Aβ, which is also present in white matter (Iwamoto et al., 1997), could negatively impact myelin-axon coupling, thereby creating a vicious cycle (Mot et al., 2018).

Another neurodegenerative disease to which oligodendroglial metabolic dysfunction appears to contribute is ALS, characterized by the progressive loss of motor neurons in the cerebral cortex and spinal cord, leading to muscle atrophy and respiratory paralysis 3–5 years after the onset of initial symptoms. However, ALS is now considered a “non-cell autonomous” disease in which glial cells may contribute to disease progression by inducing or exacerbating damage to motor neurons (Philips and Rothstein, 2014). In fact, alterations in white matter occur in both animal models and ALS patients, leading to the hypothesis that oligodendroglial dysfunction is a relevant factor in the pathogenesis of the disease (Raffaele et al., 2021). Indeed, oligodendroglial inclusions of mutated proteins associated with ALS like TDP-43, FUS and SOD1 have been found in numerous cases of both sporadic and familial ALS (Mot et al., 2018). Although these aberrant proteins are expressed ubiquitously, they particularly affect motor neurons, potentially due to the high energy demands of these cells. Thus, these inclusions could physically disrupt the transport and diffusion of metabolites such as lactate from OLs to axons by obstructing myelinic channels (Mot et al., 2018). More specifically, loss of OLs appears in the spinal cord of SOD1-G93A mice, an animal model of ALS, prior to the onset of symptoms indicative of motor neuron degeneration (Kang et al., 2013; Philips et al., 2013). These OLs are replaced by new OPCs that, however, exhibit reduced expression of key maturation proteins such as myelin basic protein (MBP) and monocarboxylate transporter 1 (MCT1), resulting in demyelination (Philips et al., 2013). Furthermore, since MCT1 is a key transporter for oligodendrocyte-axon metabolic coupling (Lee et al., 2012), OLs expressing SOD1-G93A would have their capacity to provide trophic support to associated axons limited, thereby accelerating disease progression (Mot et al., 2018).

On the other hand, the primary histopathological hallmark of multiple system atrophy (MSA)—a rare and aggressive neurodegenerative disorder—is the presence of cytoplasmic inclusions of *α*-synuclein (α-Syn) aggregates predominantly within oligodendrocytes, forming glial cytoplasmic inclusions (GCIs). MSA shares many features with other synucleinopathies, such as Parkinson’s disease. Most cases of MSA are sporadic, although a mutation in the *COQ2* gene, which encodes the enzyme responsible for coenzyme Q10 synthesis, has been linked to the disease in a Japanese patient cohort (Poewe et al., 2022). Although α-Syn accumulation does not lead to significant oligodendroglial cell death, it induces demyelination, iron overload, and disruption of autophagy, eventually triggering severe neuronal loss (Poewe et al., 2022). It remains unclear whether OLs themselves pathologically upregulate α-Syn expression within the brain or if they uptake α-Syn secreted by neurons in MSA. Furthermore, astroglial and neuronal inclusions, as well as axonal dysfunction and neuronal degeneration, are also observed; however, preclinical and postmortem studies indicate that MSA is primarily an oligodendrogliopathy (Han et al., 2022). Among the candidates responsible for oligodendroglial dysfunction is tubulin polymerization-promoting protein (TPPP/p25α), which is specific to OLs and present in GCIs (Kovács et al., 2004). Under normal conditions, this protein colocalizes with myelin basic protein (MBP), but in MSA brains, it relocates to the cell body (Song et al., 2007) and contributes to the formation of an oligodendrocyte-specific α-Syn strain with high neurodegenerative potential (Ferreira et al., 2021). Interestingly, TPPP/p25α has been reported to colocalize with mitochondrial proteins in the cytoplasm of OLs in both sporadic MSA patients and a familial MSA patient carrying the *COQ2* gene mutation (Ota et al., 2014). Additionally, it was previously observed that α-Syn expression specifically in OLs, rather than in neurons, leads to a decrease in brain levels of glial-derived neurotrophic factor (GDNF) (Ubhi et al., 2010). Given that neurons undergo degeneration in MSA, these findings suggest that mitochondrial dysfunction may induce the relocation of TPPP/p25α within OLs and a pathological increase in α-Syn expression, resulting in impaired trophic support from OLs to neurons (Mot et al., 2018).

In summary, substantial evidence suggests that metabolic dysfunction in OLs significantly contributes to the pathogenesis of various neurodegenerative diseases, including MS, AD, ALS and MSA. The age-related deterioration of myelin can disrupt metabolic coupling between OLs and axons, depriving neurons of vital trophic support and resulting in axonal damage and neurodegeneration. Consequently, enhancing myelin-axon metabolic coupling represents a promising therapeutic target for future research in neurodegeneration.

## 5 Therapeutic implications and future directions

As our understanding of OL myelin production and metabolic regulation deepens, new strategies are emerging to enhance neuroprotection and address the metabolic dysfunctions linked to neurodegenerative diseases (Figure 2). Recent advances emphasize the potential for targeted interventions aimed at correcting these dysfunctions (Cunnane et al., 2020; Han et al., 2022). Neurodegenerative disorders are challenging to treat due to their progressive and multifactorial nature. However, these diseases share common features, including systemic neuron loss in the motor, sensory, and cognitive systems, leading to a spectrum of symptoms and metabolic changes in brain energy regulation (Procaccini et al., 2016). Currently, no disease-modifying therapies can reverse or halt disease progression. Existing treatments primarily manage symptoms, highlighting the urgent need for therapies that target the underlying neuropathogenesis (Cleland et al., 2021).

Remyelination therapies for MS aim to promote the repair or formation of new myelin by using various drugs and approaches (Kantarci, 2019; Lassmann, 2018). Several compounds, such as benztropine, clemastine, and quetiapine, have been shown to enhance OL recruitment, survival, and differentiation by targeting receptors like muscarinic acetylcholine (M1) and histamine (H1, H3) (Jiang et al., 2011; Kremer et al., 2019; Münzel and Williams, 2013). Opicinumab, a monoclonal antibody against LINGO-1, also shows promise by promoting remyelination, though clinical trial results have been mixed (Lassmann, 2018; Levin and Kaplan, 2017). While remyelination is effective in early MS stages, cell depletion and inflammation in advanced stages limit its success (He et al., 2021).

Recent research highlights the potential of targeting monocarboxylate transporters (MCTs) to enhance neuroprotection in diseases like AD, MS, and ALS, where axonal integrity is compromised (Jha and Morrison, 2018; López-Muguruza and Matute, 2023). Inhibition of MCT1 in OLs leads to axonal damage, but lactate supplementation can reverse this. Furthermore, boosting MCT1 in OLs and MCT2 in neurons has been shown to prevent axonal degeneration, improve energy metabolism, and reduce oxidative stress, demonstrating potential in neurodegeneration models (Villoslada, 2016; Wu et al., 2023).

Ketogenic diets, which are high in fat and low in carbohydrates, elevate ketone body production, providing an alternative energy source for the brain (Tao et al., 2022). In neurodegenerative diseases like MS, ketogenic diets have shown promise in reducing oxidative stress and inflammation, both of which contribute to OL dysfunction (Storoni and Plant, 2015). Studies suggest that ketogenic diets reduce neuroinflammation and promote the conversion of M1 microglia to a protective M2 phenotype in experimental MS models, leading to improved motor function and decreased demyelination (Sun et al., 2023). Moreover, ketogenic diets enhance mitochondrial function, which is crucial for maintaining energy homeostasis in the brain and supporting OLs in their myelin production (Yang et al., 2019).

Lipids, particularly fatty acids, are recognized for their complex roles in neurodegenerative diseases. Short-chain fatty acids (SCFAs), produced by gut microbiota from dietary fiber (Kousparou et al., 2023), have been linked to a reduction in amyloid-beta accumulation, a hallmark of AD (Wysoczański et al., 2016). Sodium butyrate, a well-studied SCFA, has shown promise in improving cognitive and memory performance in AD models (Dyall, 2015). Medium-chain fatty acids (MCFAs), found in dairy and coconut oil (Avallone et al., 2019), act as agonists of peroxisome proliferator-activated receptors (PPARs), which influence brain function by enhancing insulin sensitivity and modulating inflammation, particularly in diseases like PD and MS (Chang et al., 2015).

Long-chain fatty acids (LCFAs) are essential for brain function, but their effects vary depending on the type and concentration. Polyunsaturated fatty acids (PUFAs), such as omega-3 (e.g., DHA and EPA) and omega-6 fatty acids, show neuroprotective properties in managing neurodegenerative diseases (Kousparou et al., 2023; Wysoczański et al., 2016). These PUFAs help regulate inflammation, protect against oxidative stress, and stabilize neuronal membranes (Dyall, 2015). For example, DHA has been shown to reduce neuroinflammation and preserve synaptic integrity, which is crucial in AD and PD (Avallone et al., 2019; Chang et al., 2015). Their effects stem from their ability to modulate immune responses and promote membrane fluidity, aiding neuronal resilience during neurodegeneration (Giacobbe et al., 2020; Yang et al., 2011).

Conversely, saturated LCFAs like palmitic acid (PA) are linked to neurotoxicity through the activation of inflammatory pathways, increasing neuroinflammation— a hallmark of neurodegenerative diseases (Vesga-jiménez et al., 2022). PA activates toll-like receptors (TLRs) and the NF-κB pathway, which leads to oxidative stress and cellular dysfunction (Osorio et al., 2020). In contrast, monounsaturated fatty acids like oleic acid (OA) have neuroprotective effects (Urso and Zhou, 2021). OA stabilizes cell membranes, supports axonal growth, and counteracts PA-induced inflammation. It also helps maintain mitochondrial health and lipid homeostasis, supporting neural repair and reducing neurodegenerative damage (Beaulieu et al., 2021; Eynaudi et al., 2021; Urso and Zhou, 2021).

## 6 Conclusion

The use of omics technology during the last decade has substantiated the pioneering ideas of del Río-Hortega about the functional role of oligodendrocyte heterogeneity in axon-myelin unit and beyond. In particular, we now know how newly formed oligodendrocytes handle calcium homeostasis during maturation; that perivascular oligodendroglia contributes to BBB formation; and that oligodendrocytes and their progenitors are immunocompetent during inflammation (Figure 2). This opens the gate to the development of therapeutic interventions targeting directly oligodendrocytes and myelin to ameliorate neurodegenerative and demyelinating diseases.

In turn, recent research grounded Río-Hortega’s ideas about oligodendroglia as a provider of trophic/metabolic support to axons exchanging glucose metabolic derivatives through monocarboxylate transporters. Strikingly, indirect evidence shows that oligodendroglia may provide myelin-derived fatty acids to support axonal function during hypoglycaemia (Asadollahi et al., 2024).

On the other hand, oligodendrocyte dysfunction may contribute to AD pathology onset and progression as amyloidosis occurs in oligodendrocyte-specific mutations that impair myelin integrity. Likewise, oligodendroglial inclusions of mutated proteins associated with ALS may disrupt the transport and diffusion of metabolites to axons prior to the onset of symptoms. Moreover, MSA oligodendropathy includes the formation of an oligodendrocyte-specific *α*-Syn strain with high neurodegenerative potential. Finally, myelin aging can disrupt metabolic coupling between OLs and axons resulting in axonal neurodegeneration. Consequently, enhancing myelin-axon metabolic coupling represents a promising therapeutic target for future research in neurodegeneration. This includes boosting MCT1 to support energy metabolism, ketogenic and fatty acid diets to reduce neuroinflammation and demyelination as well as enhancing mitochondrial function to help OLs maintain myelin production and preserve axonal integrity.
